# Priority Effects Favor Invasive *Bidens frondosa* over Its Native Congener *Bidens biternata*, While Late Arrival Incurs Higher Costs

**DOI:** 10.3390/plants14162515

**Published:** 2025-08-13

**Authors:** Chunqiang Wei, Saichun Tang, Xiangqin Li, Yumei Pan, Longwu Zhou

**Affiliations:** Guangxi Key Laboratory of Plant Conservation and Restoration Ecology in Karst Terrain, Guangxi Institute of Botany, Guangxi Zhuang Autonomous Region and Chinese Academy of Sciences, Guilin 541006, China; wcq@gxib.cn (C.W.);

**Keywords:** timing of arrival, *Bidens frondosa*, invasive plants, nitrogen addition, plant growth, species competition

## Abstract

Priority effects—the phenomenon where early-arriving species influence the establishment, growth, and reproduction of later-arriving species during community assembly—play a critical role in determining the invasion success of exotic species. However, how priority effects are influenced by nitrogen (N) availability remains understudied. The invasive species *Bidens frondosa* has rapidly expanded its range in China over the past few years. Yet it remains unclear how priority effects in *B. frondosa* versus native species may mediate invasion success, as well as how nutrient levels may alter these effects. Addressing these questions is essential for understanding the mechanisms driving *B. frondosa* invasion and for developing effective management strategies. In a greenhouse experiment, we manipulated the planting order of *B. frondosa* and its native congener *B. biternata*, then measured the growth and competitiveness of *B. frondosa* and *B. biternata* in both control and N addition treatments. Planting order greatly impacted the growth and competitiveness of both *B. frondosa* and *B. biternata*. Early arrival had more positive effects on *B. frondosa* than *B. biternata*, while late arrival more strongly inhibited *B. frondosa* than *B. biternata*. For *B. frondosa*, priority effects lessened with nitrogen addition, but the opposite occurred for *B. biternata*. Thus, priority effects may favor *B. frondosa* invasion, while late arrival, particularly under nitrogen addition, may curb its spread. As such, sowing early-germinating native species represents a useful management strategy for controlling *B. frondosa* invasions.

## 1. Introduction

Invasive plant species frequently outcompete native plants, thereby reducing biodiversity. Understanding the mechanisms behind successful invasions is crucial for controlling invasive species and restoring affected communities [1,2]. Compared to their native counterparts, invasive plant species often exhibit higher resource capture and utilization capabilities, competitiveness, and reproductive rates [3,4,5]. Furthermore, invasive species often have earlier emergence dates, higher germination rates, and faster growth rates [6,7,8]. These characteristics may enable invasive plants to successfully establish themselves in new environments.

Priority effects represent a type of phenological niche separation, in which early-arriving species influence the establishment, growth, and reproduction of species that arrive later during community assembly [9,10,11]. Priority effects can influence plant species’ relationships and community composition [11,12,13]. Early-arriving species can reduce the resources (e.g., nutrients, water, and space) available for late-arriving species and thereby negatively impact their growth [14,15]. Alternatively, early-arriving species may modify the types of niches available for late-arriving species, such as via allelopathy; these modifications can have either positive or negative effects on later-arriving species [9,16]. Currently, most experimental studies examining priority effects (i.e., those manipulating the timing of species’ arrivals) have been conducted in temperate ecosystems, with far fewer studies of tropical and subtropical ecosystems [11]. The mechanisms underlying priority effects may depend on local environmental conditions and the relationships among species, as well as the ability of study species to adapt to environmental changes [17,18,19]. Plant phenology differs between tropical/subtropical climates and temperate climates, and the key factors influencing plant phenology also differ [20]. Whether the role of priority effects in tropical and subtropical ecosystems is different from that in temperate ecosystems remains unclear.

Priority effects are recognized as an important driver of invasion success for exotic species [21,22,23]. In most cases, priority effects provide greater benefits and impose fewer costs on invasive versus native species [11,24]. For example, Dickson et al. (2012) [25] found that priority effects can result in the formation of near-monocultures by invasive species, whereas early-arriving native species did not exhibit this tendency. Ferenc et al. (2021) [26] also found that arriving early significantly increases the biomass of alien species but not native species. However, this is not a universal finding, with other studies showing no difference in priority effects between invasive and native species [19,27]. In addition, Stuble and Souza (2016) [19] found that arriving late decreased the growth of native species more than invasive species. In contrast, a recent meta-analysis described significant reductions in the performance of invasive species that arrived late, but this was not seen for native species [23]. These discrepancies among studies might stem from differences among study species and systems [19]. To better understand how priority effects shape invasion success and resistance to invasion, both the potential advantages of arriving early and the disadvantages of arriving late must be explored across a wider array of invasive and native species, as well as study ecosystems [19].

The magnitude of priority effects can vary in response to environmental conditions [28,29]. For example, resource supplementation can strengthen priority effects, enabling early-arriving species to secure more resources [28]. In highly fertile soils, competitively inferior species may therefore achieve community dominance by arriving first [30]. Similarly, priority effects allow early-arriving species to gain additional resources and produce more biomass when soil nutrient levels are high but not when they are low [28]. Anthropogenic nitrogen (N) deposition increases the availability of soil N, a crucial nutrient for plant growth in natural ecosystems. Higher soil N levels due to human activities can therefore promote the growth of invasive species and enhance their competitive ability [5,31,32]. However, whether N addition also enhances the magnitude of priority effects for invasive species remains unknown.

*Bidens frondosa*, native to North America, has rapidly invaded natural ecosystems in China, where it is classified as a first-grade invasive plant species [33]. Aqueous extracts from *B. frondosa* have been shown to inhibit seed germination and seedling growth in *Ageratum conyzoides* and *Plantago virginica* [34]. Additionally, *B. frondosa* exhibits phenotypic plasticity in response to light, nitrogen, and water availability [35,36], produces a large number of seeds [4], and has a high germination rate [7]. In Guangxi Province of China, *Bidens frondosa* is sympatric with its native congener *Bidens biternata*, and both can be found in disturbed areas, such as rural roadsides and abandoned lands. Both species are annual herbs in the Asteraceae family and share similar flowering and seed-setting periods, typically from August to October. In subtropical areas, we have observed that the emergence of *B. frondosa* and *B. biternata* seedlings occurs from late March to early May. Species with similar functional traits are more likely to compete for resources [37,38]. However, it is unclear whether priority effects have contributed to the invasion success of *B. frondosa*, such as by positively affecting its growth and competitiveness.

In this study, we evaluated priority effects on the growth (i.e., total biomass, root/shoot (*R/S*) ratio, and reproductive ratio) and competitiveness (i.e., relative dominance index (*RDI)* and relative interaction index (*RII*)) of the invasive *B. frondosa* and its native congener *B. biternata*. We also examined how exogenous N addition impacted the strength of priority effects for both species. In our greenhouse experiment, we manipulated the timing of arrival via three planting order treatments: (1) T_0_—both species sown simultaneously; (2) T_1_—*B. frondosa* sown three weeks before *B. biternata* (i.e., *B. frondosa* given priority); and (3) T_2_—*B. biternata* sown three weeks before *B. frondosa* (i.e., *B. biternata* given priority). We simulated N deposition by adding N fertilizer to a subset of replicates.

We hypothesized that (1) priority planting will confer greater benefits on the invasive *B. frondosa* than on its native congener *B. biternata*; (2) arriving late will have less of a negative impact on the invasive species than the native congener; and (3) N addition will enhance the priority effects of *B. frondosa* to a greater extent than in the native congener.

## 2. Results

### 2.1. Growth Comparison of Invasive B. frondosa and Native B. biternata

**Figure 1:**
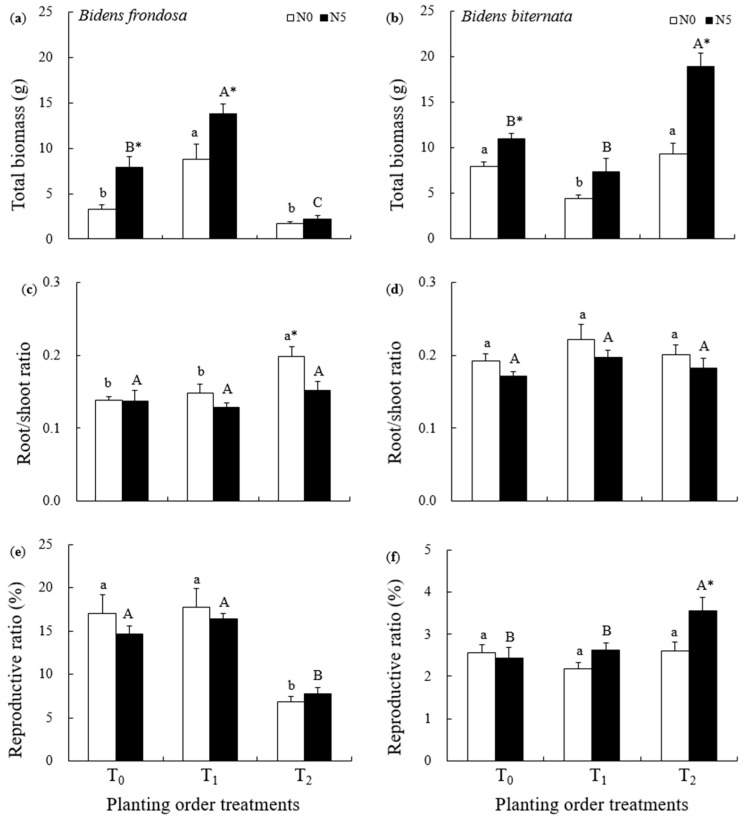
Growth of *Bidens frondosa* (Left column) and *Bidens biternata* (Right column) under different planting order scenarios with supplemental N addition (N_5_) and without N addition (N_0_). (**a**) Total biomass of *B. frondosa*; (**b**) Total biomass of *B. biternata*; (**c**) Root/shoot ratio of *B. frondosa*; (**d**) Root/shoot ratio of *B. biternata*; (**e**) Reproductive ratio of *B. frondosa*; (**f**) Reproductive ratio of *B. biternata*. (T_0_: *B. frondosa* and *B. biternata* were sown at the same time; T_1_: *B. frondosa* was sown before *B. biternate*; and T_2_: *B. biternata* was sown before *B. frondosa*. Different lowercase letters (a, b) denote significant differences among T_0_, T_1_, and T_2_ without N addition, while different uppercase letters (A–C) indicate significant differences among T_0_, T_1_, and T_2_ with N addition; stars (*) indicate significant differences between N addition and control (no fertilizer) treatments for the same planting scenario, *p* < 0.05.)

Planting order (i.e., the timing of arrival), N addition, and their interaction significantly affected the total biomass of both *B. frondosa* and its native congener *B. biternata* (Table 1). For *B. frondosa*, as compared to T_0_ (both species sown simultaneously), the total biomass increased by 74.1% (with N addition) and 167.3% (without N addition) in T_1_ (*B. frondosa* given priority) (Figure 1a). The total biomass decreased by 72.7% in T_2_ (*B. biternata* given priority) under N addition, when compared to T_0_ (Figure 1a). *B. frondosa* grown without supplemental N produced 49.2% less biomass in T_2_ than in T_0_, though this difference was not statistically significant (Figure 1a). For *B. biternata*, as compared to T_0_, the total biomass increased by 73.4% (with N addition) and 17.7% (without N addition) in T_2_, but no significant differences were observed between T_0_ and T_2_ in the absence of supplemental N (Figure 1b). The total biomass decreased by 32.9% (with N addition) and 44.9% (without N addition) in T_1_, when compared to T_0_, though the differences were not statistically significant between T_0_ and T_1_ under N addition (Figure 1b).

Planting order and N addition both affected the *R/S* ratio of *B. frondosa*, but not that of *B. biternata* (Table 1). The *R/S* ratio of *B. frondosa* grown without supplemental N was greater in T_2_ (0.20 ± 0.01) than in T_0_ (0.14 ± 0.01) or T_1_ (0.15 ± 0.01) (Figure 1c). In addition, in T_2_, the *R/S* ratio of *B. frondosa* was higher in the absence of supplemental N (0.20 ± 0.01) than under N addition (0.15 ± 0.01) (Figure 1c). The *R/S* ratio of *B. biternata* did not differ among T_0_, T_1_, and T_2_ (Figure 1d).

Planting order affected the reproductive ratio of both *B. frondosa* and *B. biternata*, while N addition only affected the reproductive ratio of *B. biternata* (Table 1). The reproductive ratio of *B. frondosa* was lower in T_2_ (7.74 ± 0.72% with N and 6.8 ± 0.67% without N addition) than in T_0_ (14.63 ± 0.92% with N and 17.05 ± 2.19% without N addition) or T_1_ (16.45 ± 0.62% with N and 17.70 ± 2.25% without N addition) (Figure 1e). The reproductive ratio of *B. biternata* was greater in T_2_ (3.55 ± 0.32%) than in T_1_ (2.63 ± 0.17%) or T_0_ (2.42 ± 0.27%) under N addition (Figure 1f). In addition, in T_2_, the reproductive ratio of *B. biternata* was greater under N addition (3.55 ± 0.32%) than when unfertilized (2.60 ± 0.21%) (Figure 1f).

### 2.2. Competitive Performance of B. frondosa and B. biternata

**Figure 2:**
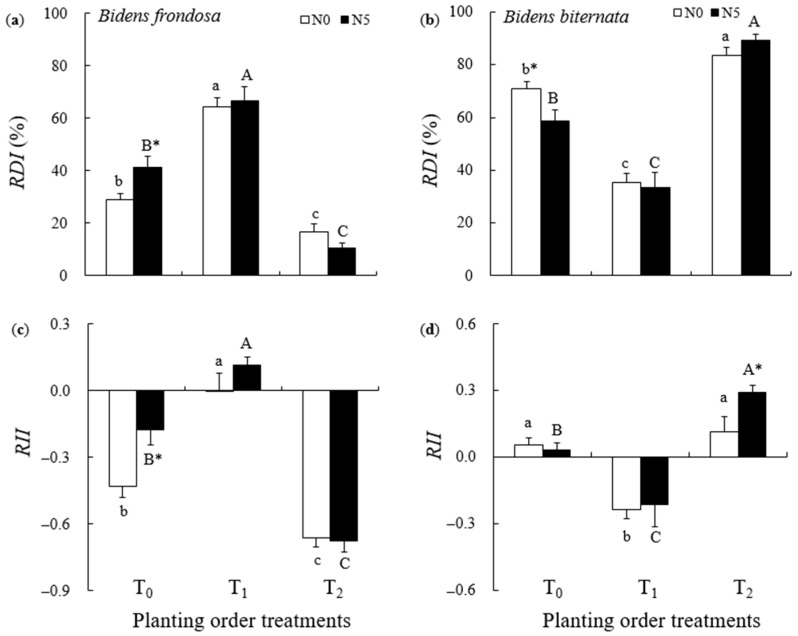
Relative dominance index (*RDI*) and relative interaction index (*RII*) for *Bidens frondosa* (Left column) and *Bidens biternata* (Right column) under different planting order scenarios with supplemental N addition (N_5_) and without N addition (N_0_). (**a**) *RDI* of *B. frondosa*; (**b**) *RDI* of *B. biternata*; (**c**) *RII* of *B. frondosa*; (**d**) *RII* of *B. biternata*. (T_0_: *B. frondosa* and *B. biternata* were sown at the same time; T_1_: *B. frondosa* was sown before *B. biternate*; and T_2_: *B. biternata* was sown before *B. frondosa*. Different lowercase letters (a–c) denote significant differences among T_0_, T_1_, and T_2_ without N addition, while different uppercase letters (A–C) indicate significant differences among T_0_, T_1_, and T_2_ with N addition; stars (*) indicate significant differences between the N addition and control treatments for the same planting scenario, *p* < 0.05.)

Planting order affected both the *RDI* of *B. frondosa* and *B. biternata* (Table 1). As compared to T_0_, the *RDI* of *B. frondosa* increased by 61.3% (with N addition) and 123.2% (without N addition) in T_1_, but decreased by 74.5% (with N) and 42.8% (without N) in T_2_ (Figure 2a). For *B. biternata*, as compared to T_0_, the *RDI* increased by 52.4% (with N) and 17.4% (without N) in T_2_, but decreased by 43.1% (with N) and 50.1% (without N) in T_1_ (Figure 2b).

Planting order also affected the *RII* of both *B. frondosa* and *B. biternata,* while N addition only affected the *RII* of *B. frondosa* (Table 1). The *RII* of *B. frondosa* was greater in T_1_ (0.11 ± 0.4 with N and 0.00 ± 0.08 without N addition) than in T_2_ (−0.68 ± 0.05 with N and −0.66 ± 0.04 without N addition) or T_0_ (−0.17 ± 0.07 with N and −0.43 ± 0.05 without N addition) (Figure 2c). Under N addition, the *RII* of *B. biternata* was greater in T_2_ (0.29 ± 0.03) than in T_1_ (−0.21 ± 0.10) or T_0_ (0.03 ± 0.03). When no exogenous N was applied, the *RII* of *B. biternata* was also higher in T_2_ (0.11 ± 0.07) and T_0_ (0.05 ± 0.03) than in T_1_ (−0.24 ± 0.04) (Figure 2d).

## 3. Discussion

We examined priority effects on the growth and competitive performance of a subtropical invasive species, *Bidens frondosa*, and its native congener, *Bidens biternata*, in a greenhouse experiment including a nitrogen addition treatment. We found that early arrival conferred greater benefits to *B. frondosa* than to *B. biternata*, supporting our first study hypothesis. However, arriving late also incurred higher costs for *B. frondosa* than for *B. biternata*, contrary to our second study hypothesis.

### 3.1. Priority Effects on Growth in the Invasive B. frondosa and Its Native Congener

In our study, *B. frondosa* exhibited a greater percent increase in biomass when sown first as compared to *B. biternata*. This suggests stronger priority effects for *B. frondosa* than *B. biternata*, supporting our first study hypothesis. This finding is also consistent with several studies conducted in temperate regions, which showed that invasive plant species benefit more from priority effects than native species [24,25,26]. One possible explanation for this pattern is that invasive species generally have higher relative growth rates (*RGRs*) [39] and tend to grow rapidly in the absence of competition from other plants. In support of this explanation, in a previous study, we found that the *RGR* of *B. frondosa* was higher than that of *B. biternata* under favorable light and water conditions [40]. Having a higher *RGR* may enable *B. frondosa* to capture more light and ultimately grow larger than *B. biternata*.

Invasive plants often display rapid growth, strong competitive ability, and tolerance of resource-limited environments [41,42,43]; these characteristics may reduce the cost of arriving late and facilitate their successful establishment in native communities [19]. However, we found that arriving late decreased the biomass of *B. frondosa* to a greater extent than that of *B. biternata*, particularly under exogenous nitrogen addition. This suggests that *B. frondosa* incurred greater costs from arriving late than *B. biternata*. Priority effects are often mediated by niche pre-emption mechanisms, in which early-arriving species reduce the amount of resources (e.g., light, soil nutrients, and water) available to late-arriving species [9]. In an earlier study, we found that light levels significantly affect the growth of *B. frondosa*, which exhibits greater total biomass and a higher *RGR* under high versus low light conditions [40]. When planted first, *B. biternata* grew more vigorously, especially in the nitrogen addition treatment. This enhanced growth likely granted *B. biternata* a competitive advantage in capturing light resources, ultimately affecting light availability for late-arriving *B. frondosa*. We have also found that low light conditions reduce the *RGR* of *B. frondosa* to a greater extent than that of *B. biternata* [40]. Thus, the greater negative impact of arriving late for *B. frondosa*, as compared to *B. biternata*, may be due to reductions in the *RGR* under low light. This finding is inconsistent with those of previous studies in temperate regions, where arriving late imposed lower costs on alien species than natives [26]. This discrepancy among studies likely stems from differences in the study species and ecosystems [19].

Invasive plants may respond to changes in resource availability by altering biomass allocation patterns [44,45]. In the control (no N fertilizer), the *R/S* ratio of *B. frondosa* increased significantly when arriving late; this shift may enhance water and nutrient uptake under stressful conditions [46]. *Bidens frondosa* demonstrates high phenotypic plasticity in response to shifts in light, N, and water availability [36,40] as illustrated here. Additionally, arriving late reduced the reproductive ratio in *B. frondosa*. This may be related to a decrease in light availability, caused by the early arrival of *B. biternata*, which would have lowered the reproductive potential of *B. frondosa*. Ferenc et al. (2021) [26] also found that arriving late reduced the number of flowerheads in exotic species. Reductions in the reproductive ratio, along with a decrease in biomass, could negatively impact seed production, potentially lowering the invasion success of *B. frondosa* in the following season.

### 3.2. Priority Effects on Competitiveness in the Invasive B. frondosa and Its Native Congener

In community assembly, the timing of species’ arrivals significantly impacts competitive interactions between invasive and native species [47,48,49]. In this study, the *RDI* and *RII* of both *B. frondosa* and *B. biternata* decreased when planted late. This suggests that arriving first limited suppression by neighboring plants, thereby strengthening overall competitive ability. Our findings align with those of Stevens and Fehmi (2011) and Kardol et al. (2012) [28,47], who highlighted that species that arrive first are more likely to adapt to local environmental conditions and dominate the site [28,47]. Similarly, our results support the established notion that early-arriving species tend to outcompete those that arrive later [24,48,49]. Notably, when arriving early, *B. frondosa* showed a greater increase in *RDI* compared to *B. biternata*, suggesting that the priority effects for *B. frondosa* are stronger. Conversely, when planted second, the *RDI* and *RII* of both species decreased. This is consistent with previous studies that showed that the competitive ability of late-arriving species can be diminished by early-arriving ones [47,49]. Moreover, when planted second, the percentage decrease in *RDI* was more pronounced for *B. frondosa* versus *B. biternata*, and the *RII* of *B. frondosa* was lower than that of *B. biternata*. This implies that *B. frondosa* was less tolerant of competition from *B. biternata* when arriving late. Late arrival therefore incurred more significant competitive disadvantages for *B. frondosa* than *B. biternata*. This is consistent with previous findings from temperate regions, where early-arriving native species can competitively exclude invasive species [24,48].

### 3.3. The Magnitude of Priority Effects for Invasive B. frondosa and Its Native Congener Under N Addition

When exogenous N was applied, the magnitude of the beneficial effects of early arrival (i.e., enhanced biomass and *RDI*) decreased for *B. frondosa* but increased for *B. biternata*. This contradicts our third study hypothesis that N addition should amplify priority effects, particularly for the invasive *B. frondosa*. When *B. frondosa* is planted first, N addition may lessen the competitive inhibition of late-arriving *B. biternata*, increasing its growth and overall performance. This should weaken priority effects for *B. frondosa* under N addition. Several other studies have found that N addition may alleviate competition between invasive and native species [50,51]. When *B. biternata* is planted first, it may grow faster and larger when additional N is supplied, thereby more strongly shading late-arriving *B. frondosa*. This could decrease *B. frondosa* growth and thereby amplify priority effects in *B. biternata*. Similarly, early-arriving species gain access to additional resources in favorable environments, often leading to stronger priority effects in high- versus low-nutrient environments [28,49]. However, this is not a universal finding, and N addition does not always alter the strength of priority effects [52]. This may be because the strength of priority effects is mediated not only by impacts on resource levels, but also by the environmental sensitivity of early-arriving species and the overlap between competing species in terms of their resource needs [18]. *Bidens biternata* is a widespread weed in India and Japan [53,54], and it is capable of vigorous growth across diverse environments varying in light and water availability [40].

Although this study was conducted in a greenhouse, our results provide valuable insights into priority effects for the subtropical invasive species *B. frondosa* under different N regimes. *Bidens frondosa* and *B. biternata* are closely related phylogenetically, as well as being ecologically similar and sympatric in their distributions. Species with similar functional traits are more likely to compete for resources when sympatric [37,38]. Further research is needed to examine how other factors, such as water availability, moderate priority effects.

## 4. Materials and Methods

### 4.1. Study Site

This study was conducted at the Guangxi Institute of Botany (110°18′01.8″ E, 25°04′49.6″ N, 170 m a.s.l.), located in Yanshan, Guilin, Guangxi Province, China. This region features a subtropical monsoon climate, characterized by a mean annual temperature of 17.8 °C and an annual precipitation of 1742 mm. We conducted the planting order experiment in the Guangxi Institute greenhouse, under a natural photoperiod (temperature 25~35 °C and relative humidity 30~40%, recorded by a temperature and humidity meter, Beijing Honghai Yongchang Instrument Technology Development Centre, Beijing, China).

### 4.2. Plant Materials

In October 2016, we collected seeds from 20 *Bidens biternata* and 20 *Bidens frondosa* individuals growing on abandoned land within Guilin City, Guangxi, China. We stored the seeds in paper bags at room temperature. Both *B. frondosa* and *B. biternata* exhibit high seed germination rates, exceeding 90% and 75% at 20 °C, respectively [7,55].

### 4.3. Experimental Design

Our experiment was conducted in plastic pots with a diameter of 23 cm and a height of 18 cm. Each pot was filled with homogenized topsoil up to 5 cm from the top. The topsoil was obtained from an abandoned site in Guilin City. The baseline chemistry of the topsoil was as follows: the soil pH measured 7.14, organic matter 12.19 g kg^−1^, available N 117.63 mg kg^−1^, available P 29.45 mg kg^−1^, and available K 108.89 mg kg^−1^. To minimize the influence of the soil seedbank, the top 2 cm was removed before collecting topsoil to fill the experimental pots. Additionally, we regularly removed any non-target plant species (i.e., those not sown in the experiment) that emerged during the experimental period.

To establish priority treatments, we set a three-week sowing interval between *B. frondosa* and *B. biternata*, following the approach used by Ulrich and Perkins (2014) and Wilsey et al. (2015) [6,48]. For the simultaneous arrival treatment (T_0_), we sowed 10 seeds of each species into each pot on the same date. To establish priority for *B. frondosa* (T_1_), we sowed 10 *B. frondosa* seeds per pot on 28 March 2017, and allowed these to establish for three weeks. We then added 10 *B. biternata* seeds to each pot on 18 April 2017. The priority treatment for *B. biternata* (T_2_) followed the same procedure as for T_1_ but with the species reversed. Additionally, we sowed 20 seeds of each species separately in pots for use as monocultures. Plants were thinned to six plants per pot (three individuals per species in the mixtures and six individuals per species in the monocultures) after the emergence period concluded.

Nitrogen supplementation was initiated on 26 May 2017, when the seedlings of both species were well established. The average annual N wet deposition in China is 21.1 kg ha^−1^ year^−1^ [56]. As such, we tailored the N addition treatment so that experimental pots received 5 g/m^2^ N annually. NH_4_NO_3_ has been widely used to simulate atmospheric N deposition in many experiments [31]. Following He et al. (2012) [31], we applied N in the form of NH_4_NO_3_ dissolved in deionized water. A total of 0.645 g of NH_4_NO_3_ was added to each N addition pot on three dates (26 May, 3 June, and 12 June 2017). We also watered all pots daily throughout the experimental period. Each treatment included seven replicates.

In early August, we harvested all roots, shoots, leaves, and reproductive parts (i.e., inflorescences/peduncles, flowers, and fruits) from all experimental individuals; plant materials were dried at 70 °C until a constant weight and then weighed. We analyzed plant growth using the total biomass (the summed biomass of all roots, shoots, leaves, and reproductive parts), the root-to-shoot (*R/S*) ratio (root biomass/the summed biomass of all shoots, leaves, and reproductive parts), and the reproductive ratio (reproductive part biomass/total biomass).

We compared the competition performance of *B. frondosa* and *B. biternata* using the relative dominance index (*RDI*) [57] and the relative interaction index (*RII*) [58], which are based on the total biomass of each species. The *RII* was used as an estimate of the competitive response following Gruntman et al. (2013) [59]. We calculated the *RDI* and *RII* as follows:

*RDI_a_* = *B_ab_*/(*B_ab_* + *B_ba_*) × 100%

*RDI_b_* = *B_ba_*/(*B_ab_* + *B_ba_*) × 100%

*RII_a_* = (*B_ab_* − *B_a_*)/(*B_ab_* + *B_a_*)

*RII_b_* = (*B_ba_* − *B_b_*)/(*B_ba_* + *B_b_*)

where *a* and *b* represent *B. frondosa* and *B. biternata*, respectively; *B_ab_* is the total biomass of *B. frondosa* when grown with *B. biternata*; *B_ba_* is the total biomass of *B. biternata* when grown with *B. frondosa*; *B_a_* is the total biomass of *B. frondosa* in monoculture; and *B_b_* is the total biomass of *B. biternata* in monoculture. The value of *RDI* ranges between 0% and 100%, and a high *RDI* indicates that the focal species is highly competitive [60]. The value of *RII* ranges from −1 to +1, with negative values indicating suppression by neighbors and higher (i.e., less negative) values indicating greater tolerance of competition [59].

### 4.4. Data Analysis

We analyzed the growth (total biomass, *R/S* ratio, and reproductive ratio) and competitive performance (*RDI* and *RII*) of *B. frondosa* and its native congener *B. biternata* separately. We used two-way ANOVAs to test for effects of planting order, N addition, and their interaction on growth and competitive performance in each species. We used one-way ANOVAs and least-significant difference (*LSD*) post hoc tests to evaluate the effects of sowing date in *B. frondosa* and *B. biternata* individually. For each species, an independent-sample *t*-test was used to compare samples with and without N addition. All tests were considered statistically significant at *p* < 0.05. We performed all statistical analyses using SPSS 18.0 (SPSS Inc., Chicago, IL, USA).

## 5. Conclusions

In our greenhouse experiment, arriving early enhanced the growth and competitive ability of *B. frondosa* to a greater extent than for *B. biternata*. Conversely, arriving late resulted in more pronounced reductions in growth and competitive ability for *B. frondosa* versus *B. biternata*. This suggests that *B. frondosa* experienced both stronger priority effects and also greater costs for arriving late. Furthermore, control and N addition treatments differed in terms of the shifts in biomass and *RDI* associated with planting order for both species. Therefore, the strength of priority effects is likely moderated by soil nitrogen availability. Our results illustrate how arrival timing influences *B. frondosa* invasion success, offering valuable insights for its control and the restoration of invaded plant communities. Arriving early may facilitate *B. frondosa* invasion, while, conversely, the early arrival of native species can suppress *B. frondosa* growth and competitiveness. Therefore, seeding early germinating native species represents a promising strategy to curb *B. frondosa* growth, thereby reducing invasion risk.

## Figures and Tables

**Table 1:** Two-way ANOVA of the effects of time of arrival (i.e., the planting order, T) and N addition (N) on growth and competitiveness in *Bidens frondosa* and *Bidens biternata*.

Source	df	Total Biomass *F*-Value	*R/S* Ratio *F*-Value	Reproductive Ratio *F*-Value	*RDI* *F*-Value	*RII* *F*-Value
(a) *B. frondosa*						
Time of arrival (T)	2	47.04 ***	6.90 **	28.40 **	100.50 ***	82.74 ***
N addition (N)	1	18.31 ***	5.99 *	0.62	0.88	6.69 *
T × N	2	3.36 *	2.02	0.72	3.12	2.87
(b) *B. biternata*						
Time of arrival (T)	2	32.47 ***	2.19	5.23 **	100.50 ***	27.58 ***
N addition	1	37.96 ***	4.01	5.17 **	0.88	1.63
T × N	2	6.87 **	0.02	2.93	3.12	1.63

Level of significance: * *p* < 0.05, ** *p* < 0.01, *** *p* < 0.001

## Data Availability

The original contributions presented in the study are included in the article; further inquiries can be directed to the corresponding author.

## Abbreviations

The following abbreviations are used in this manuscript:

RDI	Relative dominance index
RII	Relative interaction index
RGR	Relative growth rates
